# Baseline parenchymal blood volume is a potential prognostic imaging biomarker in patients with malignant liver tumors treated with transarterial chemoembolization

**DOI:** 10.1007/s00261-024-04240-9

**Published:** 2024-04-20

**Authors:** Thomas J. Vogl, Eileen Isabell Emrich, Tatjana Gruber, Jörg Trojan, Simon Bernatz

**Affiliations:** 1 Institute for Diagnostic and Interventional Radiology, Theodor-Stern-Kai 7, 60590 Frankfurt, Germany; 2 Kurmainzerstraße 21, 61440 Oberursel, Germany

**Keywords:** Syngo Artis Zeego, Syngo Artis Pheno, Parenchymal blood volume, Transarterial chemoembolization, TACE, Liver cancer, Survival

## Abstract

**Purpose:**

To assess the prognostic value of Parenchymal Blood Volume (PBV) in predicting survival, tumor response, and PBV response after transarterial chemoembolization (TACE).

**Methods:**

A total of 137 patients with malignant liver tumors who were treated with TACE between 07/2016 and 07/2018 were evaluated. Computed tomography illustrations were reworked at a dedicated workstation to create a PBV map which was overlapped with the associated magnetic resonance image to determine tumor diameter and PBV. Patients were divided into two groups according to their initial PBV value: PBV < 50 or ≥ 50 ml/l.

**Results:**

Retrospectively, for patients with at least 2 TACE and initial PBV < 50 ml/l (*n* = 27), the tumor volume, regardless of the primary tumor type, decreased by 13.26%, and PBV showed a decrease of 23.11%. For 84 patients with PBV ≥ 50 ml/l, the tumor volume decreased by 24.01%, and PBV showed a more substantial decrease of 44.69% (both *p* < 0.001). In the overall study population (*n* = 137), patients with an initial PBV ≥ 50 ml/l (*n* = 101) survived for an average of 15.05 months, while patients with an initial PBV < 50 ml/l (*n* = 36) survived for 10.01 months (*p* < 0.002). Subgroup analysis indicated that median survival in the HCC group was longer at PBV ≥ 50 ml/l. For CRC and other primary tumors, the survival time for high and low initial PBV was almost identical.

**Conclusion:**

Our study reveals a noteworthy correlation between high initial PBV values and a significant reduction in both relative and absolute tumor volume. This association suggests a potential prognostic indicator, indicating that elevated PBV may signify a more favorable response to transarterial chemoembolization (TACE). Additionally, patients with high initial PBV values experienced an extended overall survival time. Notably, the subgroup analysis highlighted a prolonged survival time in the HCC group with elevated initial PBV values. These findings underscore the potential significance of assessing PBV as a predictive factor in the context of TACE, especially in specific tumor entities such as HCC. Further investigations are essential to validate and extrapolate these observations to optimize patient outcomes.

**Graphical abstract:**

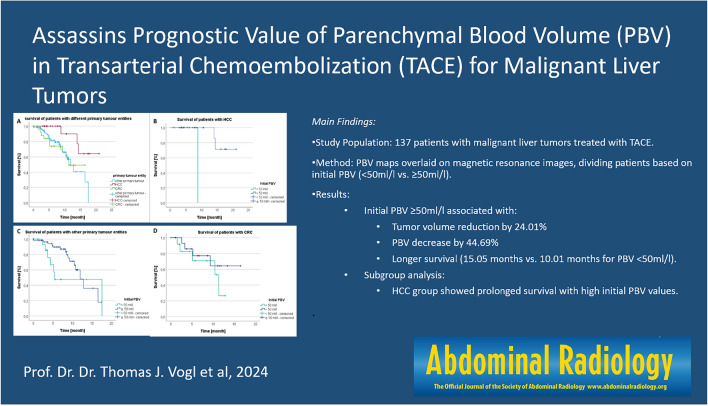

**Supplementary Information:**

The online version contains supplementary material available at 10.1007/s00261-024-04240-9.

## Introduction

Primary and secondary liver cancer are among the leading causes of death worldwide [5]. Hepatocellular carcinoma (HCC) is the most common malignant disease of the liver causing approximate 700,000 deaths per year around the world [11]. 85–95% of HCC are inoperable when they are diagnosed, leading to a treatment which is focused on interventional methods [20]. Apart from HCC, liver metastases can also originate from various other tumor types. Over 60% of colorectal carcinoma (CRC), metastasize to the liver and over 90% of the patients with CRC will die because of this metastasis [8]. Therapeutic procedures for patients with malignant liver diseases are based on the Barcelona Clinic Liver Cancer (BCLC) classification, which specifically applies to patients with HCC: in patients without vascular invasion or metastases (stage A and B), a curative approach such as surgical resection (stage 0 and A) is the preferred treatment [19]. Stage C represents a palliative therapy situation; systemic chemotherapy is considered an addition to symptomatic therapy [19]. Further established therapy options are TACE, microwave ablation (MWA), and radiofrequency ablation (RFA), which also can be used in combination [20]. TACE is used in palliative therapy approaches, but also serves as a bridge to liver transplantation or as symptomatic therapy option [13].

In TACE, arterial access is gained using the Seldinger technique, and the tumor-supplying vessels within the celiac artery are identified through angiographic examination [1]. When the catheter has reached the tumor supplying segment arteries, intraarterial injection of chemotherapeutic agents and embolizing agents may be applied [1]. This leads to an occlusion of the vessel and thus to a reduction and necrosis of the tumor tissue [21]. The dual arterial and portal venous blood supply refers to the ability of functional liver tissue to maintain viable perfusion even in the presence of occluded hepatic arteries [22]. The conventional TACE with Lipiodol was developed in the early 1980s and is until now the best TACE technique based on the unique characteristic of Lipiodol to be selectively uptaken and remain in hyperarterialyzed liver tumors [2]. The amount of Lipiodol (ml) should be at least cover the tumor diameter in cm but should not be overdosed to avoid accumulation in healthy liver tissue [23]. The embolization material EmboCept, a hydrolyzed potato starch with epichlorohydrin, has the ability to release specific amounts of an active ingredient within the tumor tissue in a delayed manner and thus facilitates the reperfusion of the area by arterial blood [23].

The response to TACE can be assessed with the RECIST and mRECIST criteria, a 2-dimensional measurement of the longest tumor diameter either by MRI or by spiral CT [18]. Additionally, C-arm CT offers the possibility to determine the parenchymal blood volume (PBV) of the tumor [14].

The parenchymal blood volume of a tumor is detectable due the administration of contrast agents in perfusion CT. The PBV maps are presented with repetitive images that can be automatically calculated and quantified using mathematical models. The 1st rotation (mask run) is followed by a 2nd rotation (fill run), where PBV data collection is performed by manual contrast agent injection into a peripheral vein (Fig. 2B) [24]. To gain an understanding of how the PBV maps are created, the technique can be found in Zellhoff et al. [27]. The created perfusion maps are automatically stored in DICOM format and can be reconstructed and edited at a especially equipped and linked workstation (Siemens Syngo X Leonardo). PBV represents a potential alternative option for assessing the response to TACE, particularly in relation to survival and tumor response.

Current publications show the assessment of the treatment course based on PBV in patients with primary and secondary liver and lung tumors [9, 10, 15, 16, 24]. These studies demonstrated PBV as a response parameter, indicating an improved response to TACE with high initial values [24, 25]. While highlighting PBV as a promising biomarker, offering insights into tumor angiogenesis, vascular perfusion, therapeutic responses, and prognostic factors, it's noteworthy that there has been no further investigation into the association between high initial PBV and prolonged patient survival to date [24, 25].

The present study therefore looks at TACE as a treatment option in patients with malignant liver metastases as well as patients with HCC as primary tumor and examines whether an initially measured high PBV-value is associated with prolonged survival and a better tumor response and whether the PBV-value can be used as a prognostic marker.

## Materials and methods

### Study design

**Fig. 1:**
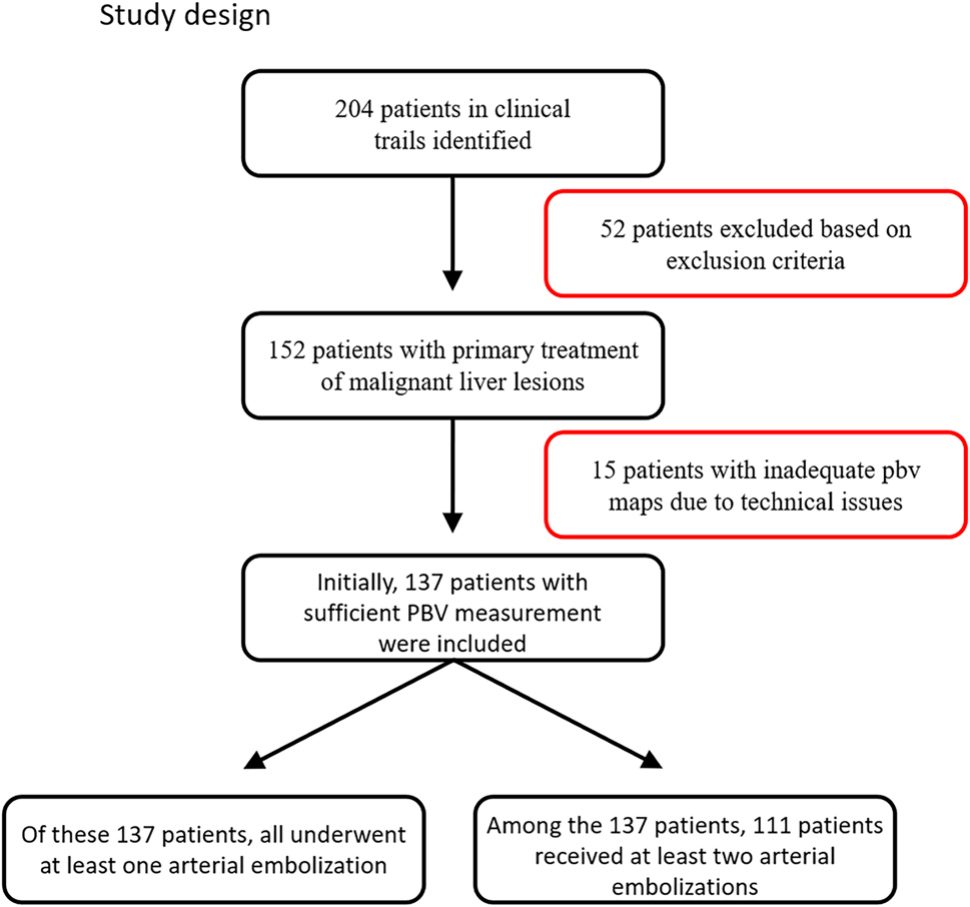
Study design

In this study, patient data were retrospectively collected and analyzed over a period of 24 months (July 2016–July 2018). Patients with malignant liver diseases who had received TACE in the institute for interventional and diagnostic radiology at the Goethe University Frankfurt were included. A consenting ethics vote was issued by the Frankfurt Ethics Committee on January 17, 2019 (business no 376/18) (Fig. 1).

### Patient characteristics

137 Patients with malignant liver diseases were included in this study. All patients are proceeded by an interdisciplinary tumor conference, in which the indication for a TACE was decided. The patient and tumor characteristics as well as the origin of malignant liver diseases, number of TACE sessions and the angiography system used can be seen in Table 1 and the primary tumor entities are displayed in Table 4. Table 2 presents the inclusion and exclusion criteria for this study. For the analysis of tumor size and PBV reduction, patients with only one TACE session were excluded. This decision was made because at least two measurements are required for such a comparison to establish a reliable basis for the analysis. This ensures that the results are based on multiple observations, providing a more accurate reflection of changes over the course of TACE treatment. However, for the survival analysis, where only one value is needed, exclusion of single sessions is not necessary.

**Table 1:** Characteristics of involved patients

Patient characteristics	All patients	PBV < 50 ml/l	PBV ≥ 50 ml/l	*p* value
Total number of patients	137	36 (26.3%)	101 (73.7%)	0.141
Sex				0.141
Male	77 (56.2%)	24 (66.7%)	53 (52.5%)	
Female	60 (43.8%)	12 (33.3%)	48 (47.5%)	
Age	Mean 61 years (range 22–86 years)	58.58 (SD 13.64 95% CI 53.97, 63.2)	61.2 (SD 12.72 95% CI 58.7, 63.71)	
Primary tumor entities				0.003
HCC	27 (19.7%)	2 (5.6%)	25 (24.8%)	
CRC	30 (21.9%)	14 (38.9%)	16 (15.8%)	
Others	80 (58.4%)	20 (55.6%)	60 (59.4%)	
Liver metastasis	110			
Tumor size (longest diameter)	Mean 48.42 mm (range 5.48–221.97 mm)			
PBV volume	Mean 49.199 ml/l (range 1.54–198.03 ml/l)			
Number of TACE sessions				
One session	137 (100%)			
Two sessions	111 (81.02%)			
Three sessions	63 (45.99%)			
Four sessions	31 (22.63%)			
Five sessions	18 (13.14%)			
Six sessions	7 (5.11%)			
Used Angiography-System	Artis Pheno Dyna-CT (59.8%)			
	Artis Zeego (40.2%)

**Table 2:** Inclusion and exclusion criteria

Inclusion criteria	Exclusion criteria
At least 18 years old	Patients who have had TACE before first PBV-measurement
Indication for TACE decided in an interdisciplinary tumor conference	Acute systemic infection
	Renal impairment with serum creatinine > 2.0 mg/dl
Mentally competent to give independent consent	Synthesis disorder of the liver with INR > 1.5 and Serum Albumin < 2.0 mg/dl
Only correct slice thickness of 7.2 mm in MRI image	

The patients were divided into three groups according to their respective tumor entity: HCC, CRC, and other primary tumors (Table 3). The ‘other’ category comprises various malignant liver diseases not specifically categorized under HCC or CRC. The diseases falling into the ‘other’ category and their respective proportions are detailed in the Table 4. 111 Patients received between 2 and 6 TACE in regularly intervals, on average 3 TACE per patient. The following chemotherapeutic agents were used: Mepivacain (regional aesthesia), 8 mg/m^2^ Mitomycin C (MedacVR, Germany, Hamburg), 1000 mg/m^2^ Gemcitabine (GemzarVR, Eli Lilly, Indianapolis, IN), and 35 mg/m^2^ Cisplatin (TevaVR, Germany, Radebeul). Further medication used: 10 ml Lipiodol (contrast media; GuerbetVR, German, Sulzbach) and 200–450 mg EmboCept (embolization agent; PharmaCept GmbH, Germany, Berlin).

**Table 3:** Primary tumor entities divided into three subgroups

	Primary tumor entities	
	Patients	%
Other primary tumor entities	80	58.4
HCC	27	19.7
CRC	30	21.9
Total	137	100

**Table 4:** Distribution of patients by primary tumor types

	Primary tumor entities	
	Frequency	Percentage
Adenoidcystical Ca	2	1.5
Choroid melanoma	7	5.1
Bronchial Ca	3	2.2
Cholangiocellular Ca	12	8.8
Ewing sarcoma	1	0.7
HCC	27	19.7
Colorectal Ca	30	21.9
Stomach cell Ca	1	0.7
Malignant melanoma	2	1.5
Mamma Ca	23	16.8
Neuroendocrine Ca	4	2.9
Oesophageal Ca	2	1.5
Ovarial Ca	5	3.6
Pancreas Ca	13	9.5
Prostate Ca	1	0.7
Urothelium Ca	1	0.7
Cervix Ca	3	2.2
Total	137	100

Only patients who received PBV measurement during TACE were eligible for the study. TACE was performed with one of the two robotic angiography systems: Syngo Dyna Artis Zeego CT Siemens (1st generation) and Syngo Artis Pheno Dyna CT Siemens (2nd generation). The system used and the technical parameters are presented in Table 5. The patient information was pseudonymized stored as associated ID and saved in Excel and SPSS tables.

**Table 5:** Technical data of 1st- and 2nd-generation systems

Parameters	First-generation system	Second-generation system
X-Ray tube	MEGALIX Cat plus	GIGALIX
Maximum tube voltage (kV)	125	125
Tube-power (kW)	80	90
Focal spot size	0.3–1.0	0.3–0.7
Detector type	Amorphous silicon flat detector as40	16-Bit crystalline silicone detector zen40HDR
Detective Quantum Efficiency	74%	79%
Resolution (p × 2)	2480 × 1920	2496 × 1856
Visible resolution	1024 × 1024	2496 × 1856
Dosimeter producer	DIAMENTOR ®, PTW GmbH, Freiburg, Germany	

### PBV measurement

**Fig. 2:**
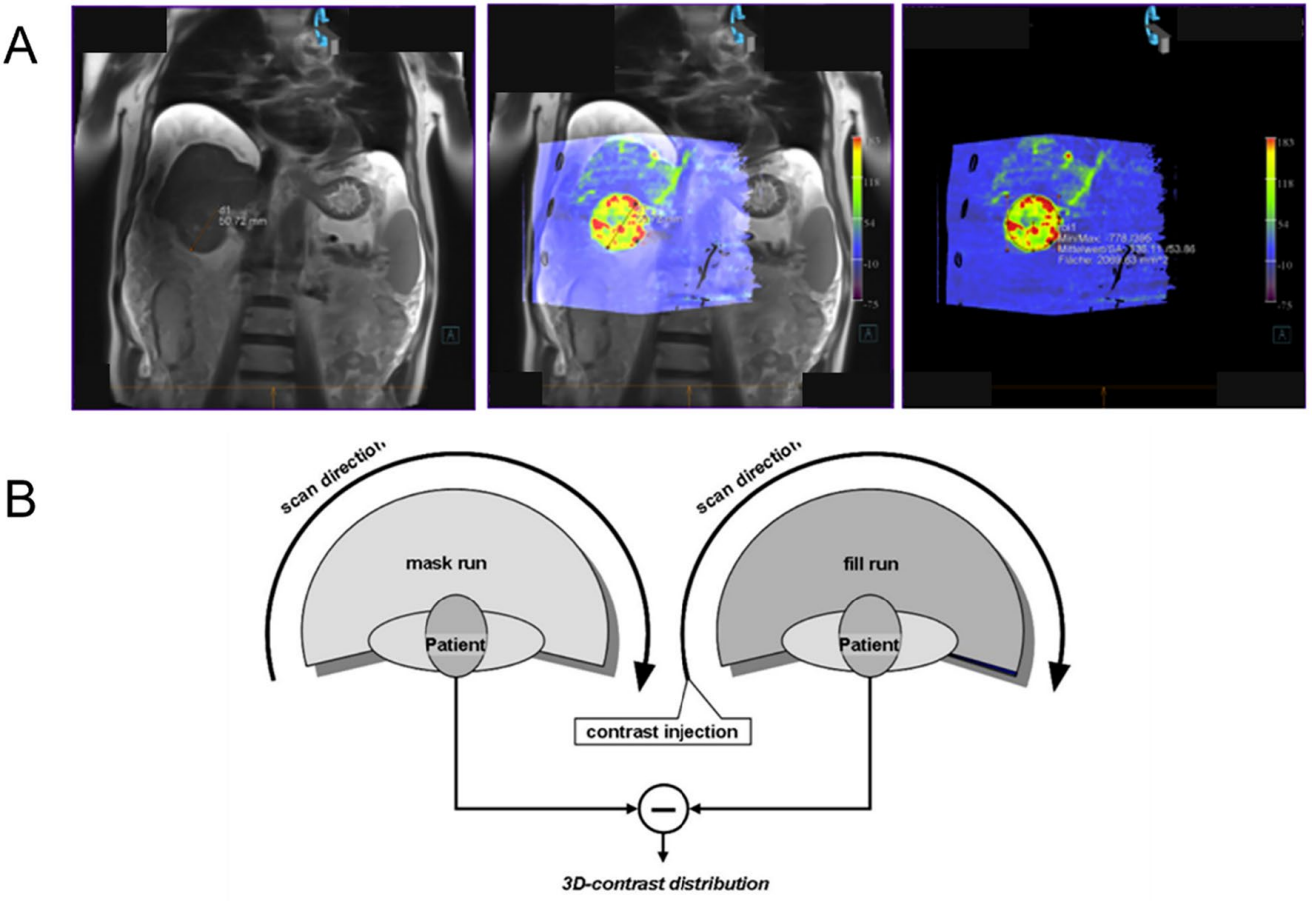
**A** Creation of a PBV image. **B** Schematic presentation of the acquisition protocol and measurement of parenchymal blood volume

The PBV value was evaluated at a specially designed workplace (Siemens Syngo X Leonardo). In our study, both PBV (Parenchymal Blood Volume) and tumor volume were measured at a single site, specifically focusing on the largest lesion for each patient. While some patients presented with multiple lesions due to different primary tumor identities, our approach aimed to assess the treatment impact on the most prominent lesion. The TACE intervention, although targeting a specific lesion for PBV and tumor volume measurements, was designed to address the entire tumor burden comprehensively. All MRI images were evaluated either in the coronal or transversal section with a slice thickness of 7.2 mm in the largest possible diameter. The MRI was fused with the CT perfusion image. The overlap allows for the automatic measurement of the region of interest (ROI) in the perfusion image, which is necessary for determining the PBV of the tumor (Fig. 2).

### Analysis and data collection

All data was analyzed using a statistical computer software (IBM SPSS, version 24). The Mann–Whitney-*U* test was used to compare the two PBV subgroups (< 50 ml/l and ≥ 50 ml/l) and to determine the significance. The tumor size difference and the PBV changes were further analyzed with linear regression and the Cox regression hazard ratio was determined. The data was analyzed by the three described tumor groups: HCC, CRC, and other primary tumors. The survival analysis was performed according to the Kaplan–Meier method. Subsequently, the results were compared with literature values.

## Results

### Reduction of tumor size and PBV

**Fig. 3:**
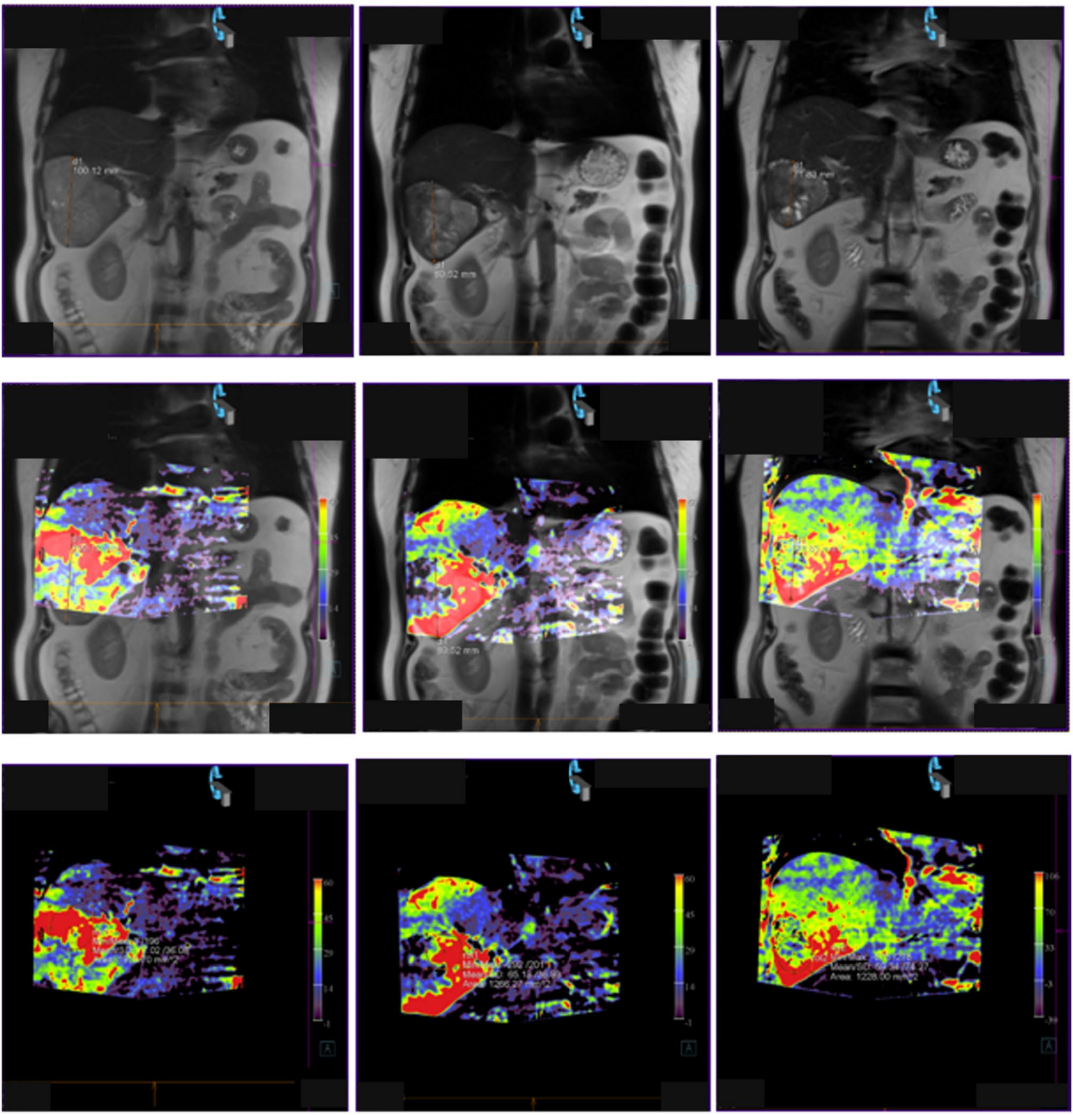
Patient with HCC after 3 TACE-sessions and 3 PBV-measurements. From left to right site you see first to third TACE. The upper row shows MRI images, in the middle row the associated fusion images are presented and generated PBV images are shown in the lower row

The tumor size and the PBV were determined during the TACE sessions. Figure 3 shows MRI and PBV images as well as fusion images of a patient with HCC after the first, second, and third TACE session as an example. The reduction of the tumor size and PBV is clearly visible.

The reduction of tumor size and PBV was initially determined from the first TACE session to the second TACE session (Supplement Fig. 1). A total of 111 patients were included, and the time period between these sessions had an average (*A*) of 33 days (range 11 to 141 days). For the 27 patients with initial PBV < 50 ml/l the tumor size was reduced by 11.01% (SD 15.83%, 95% CI [5.29, 17.50]) and PBV by 30.9% (SD 44.93%, 95% CI [15.29, 49.68]). For 84 patients with PBV ≥ 50 ml/l, the tumor volume changed by 14.16% (SD 12.27%, 95% CI [11.63, 16.69]) and PBV by 28.03% (SD 25.12%, 95% CI [22.13, 33.37]. Thus, significant differences in tumor size reduction (*p* = 0.039) and PBV reduction (*p* < 0.001) were observed between the two patient groups with PBV < 50 ml/l and ≥ 50 ml/l. The reduction in tumor size increased with a higher initial PBV ≥ 50 ml/l. The PBV after the first TACE session reduced more if the patient had a lower initial PBV with < 50 ml/l.

**Fig. 4:**
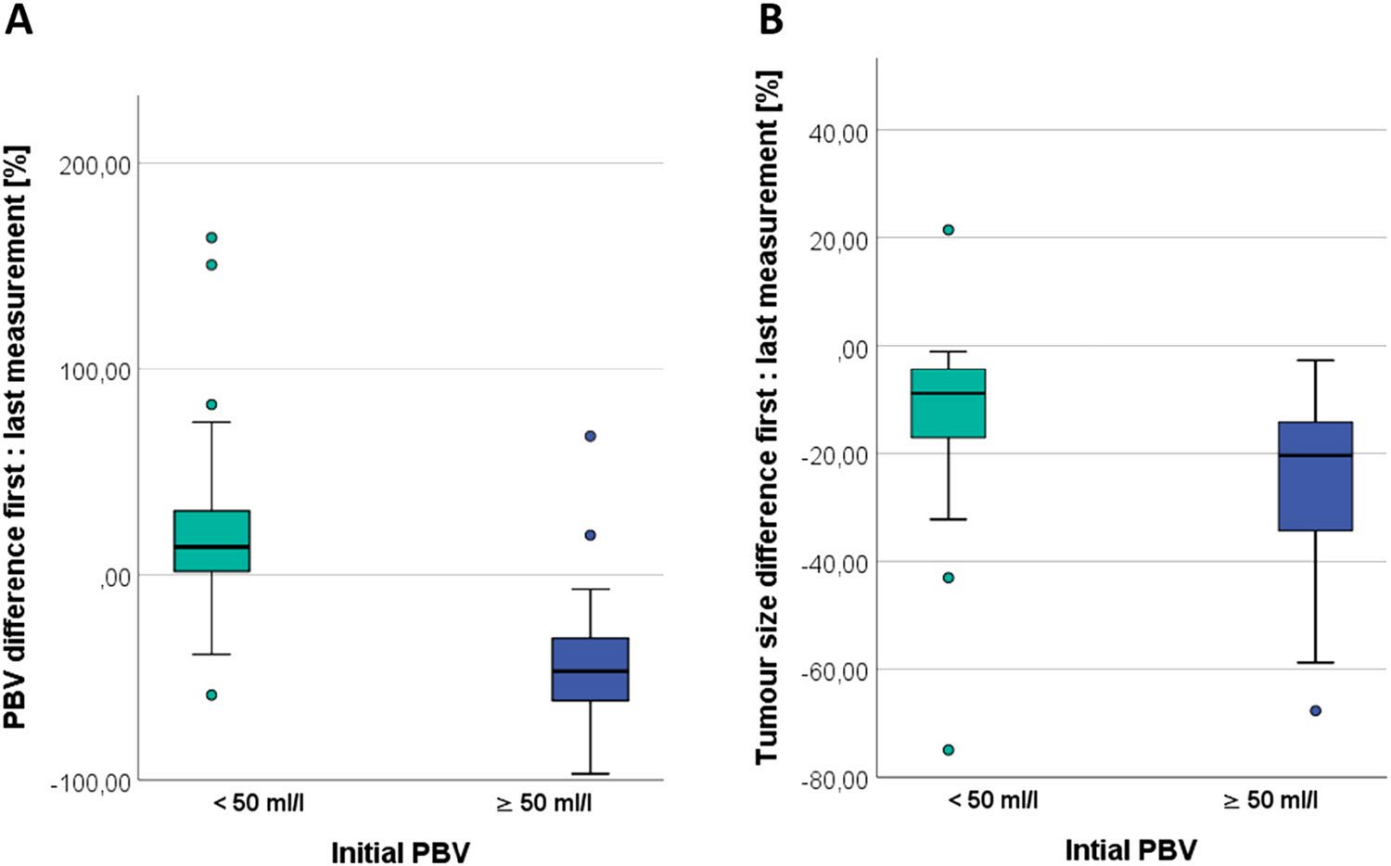
Boxplot **A** PBV differences all measurements (%) and **B** tumor size difference all measurements (%). This Box plots depicting the differences in PBV (%) between patients with initial PBV values > and < 50 ml/l (**A**) and the corresponding differences in tumor volume (%) in patients with PBV values > and < 50 ml/l (**B**). Statistical significance was observed with *p* < 0.001 for both PBV and tumor size differences

To determine the overall effect in tumor size and PBV reduction, the measurements of the first and the last TACE sessions were compared. In 111 patients, with an average of 3 TACE sessions, a correlation was demonstrated between the PBV values and the reduction of liver tumor size between the first and the last measurement. Figure 4 presents box plots illustrating the distribution of PBV values and the change in liver tumor size between the first and last measurements. This representation is based on the results of the Mann–Whitney *U*-test. The time period between the first and the last TACE session had an average (*A*) of 68 days (range 23 to 452 days). For the 27 patients with initial PBV < 50 ml/l the tumor size was reduced by 13.26% (SD 17.03%, 95% CI [7.47, 20.81]) and PBV increased by 23.11% (SD 49.69%, 95% CI [49.27, 73.89]). For 84 patients with PBV ≥ 50 ml/l, the tumor volume was reduced by 24.01% (SD 13.29%, 95% CI [21.36, 26.81]) and PBV by 44.69% (SD 25.38%, 95% CI [49.27, 73.89]. For both variables, the difference between PBV groups was significant with *p* < 0.001 for PBV (Fig. 4A) and *p* < 0.001 for the tumor size (Fig. 4B). Thus, the patient subgroup with lower initial PBV (< 50 ml/l) showed a significantly smaller reduction in tumor size and PBV compared with patients with a high initial PBV (≥ 50 ml/l). This effect was enhanced after several TACE measurements.

The linear regression analysis showed a significant association between the tumor size reduction and the PBV reduction from the first to the last TACE measurement (*p* < 0.001) with *R*^2^ 0.202.

### Survival analysis

**Fig. 5:**
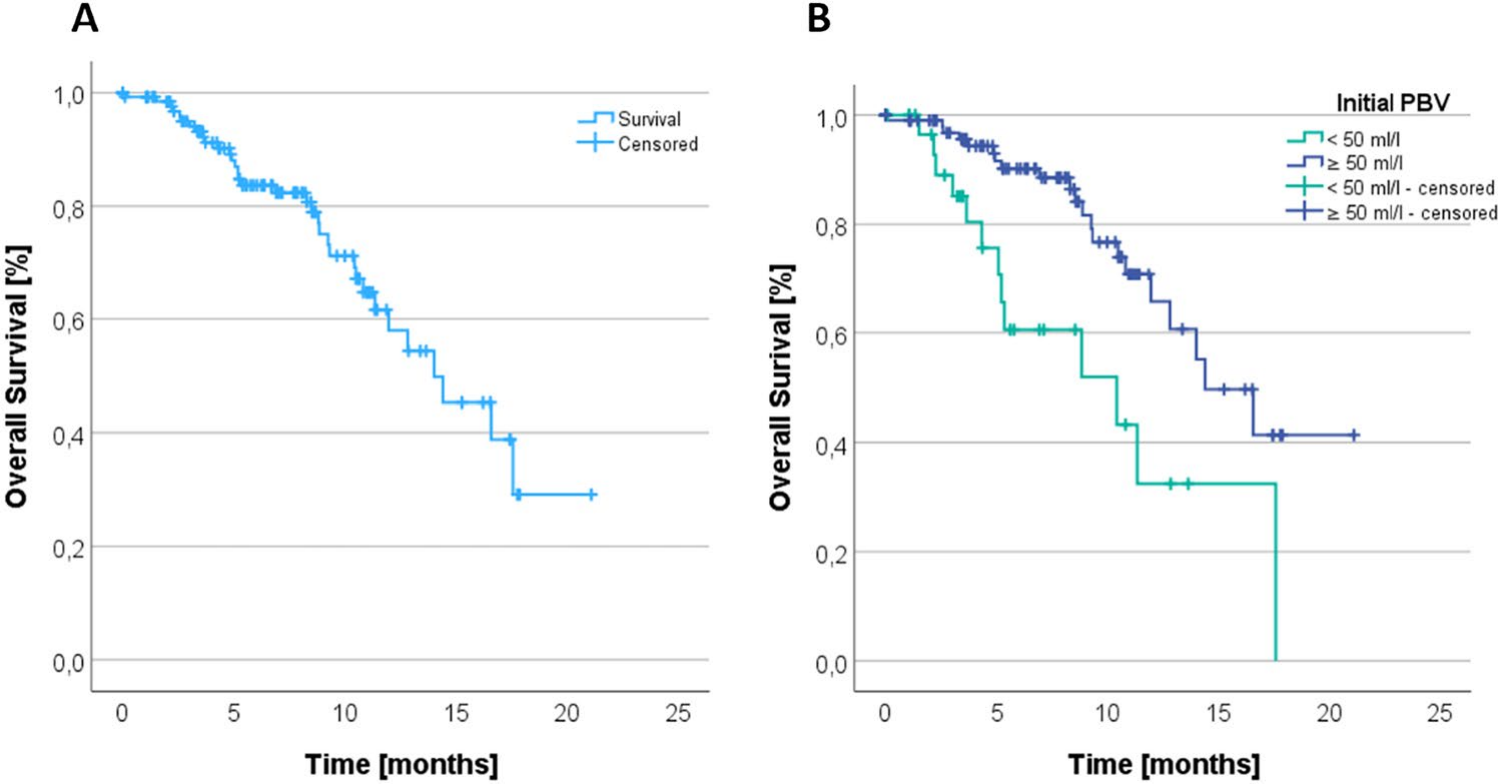
**A** Overall survival of all patients in month. **B** Difference overall survival of all patients in month related to initial PBV subgroups (< 50 ml/l and ≥ 50 ml/l) measured in the first TACE session

The overall survival time for all patients with at least one TACE session (*n* = 137) was analyzed using the Kaplan–Meier method and compared with Log-Rank statistics. The average overall survival was 13.82 months (Fig. 5A). To investigate the impact of the initial PBV on the overall survival time, the patients were again divided into the two subgroups with high initial PBV (≥ 50 ml/l) and lower initial PBV (< 50 ml/l). Patients with PBV ≥ 50 ml/l (*n* = 101) survived for an average of 15.05 months; patients with PBV < 50 ml/l (*n* = 36) survived on average 10.09 months (*p* = 0.002).

Hence, it was shown that there is an association between the initially measured PBV value and overall survival and that an initially higher PBV increased the overall survival time significantly (Fig. 5B). Additionally, the Cox regression hazard ratio was determined, i.e., the probability of dying in a certain time period. Patients with a higher initial PBV (≥ 50 ml/l) had a significantly lower risk of dying in the observed time period (*p* = 0.003), namely 65.1% (SE 35.9%, 95% CI 28.9, 82.4), even with the adjusted variables age, primary tumor entity, and sex. There was no significant linear regression between the tumor size reduction between the first and the last TACE session and the survival of the patients.

### Dependence of survival on the tumor entity

**Fig. 6:**
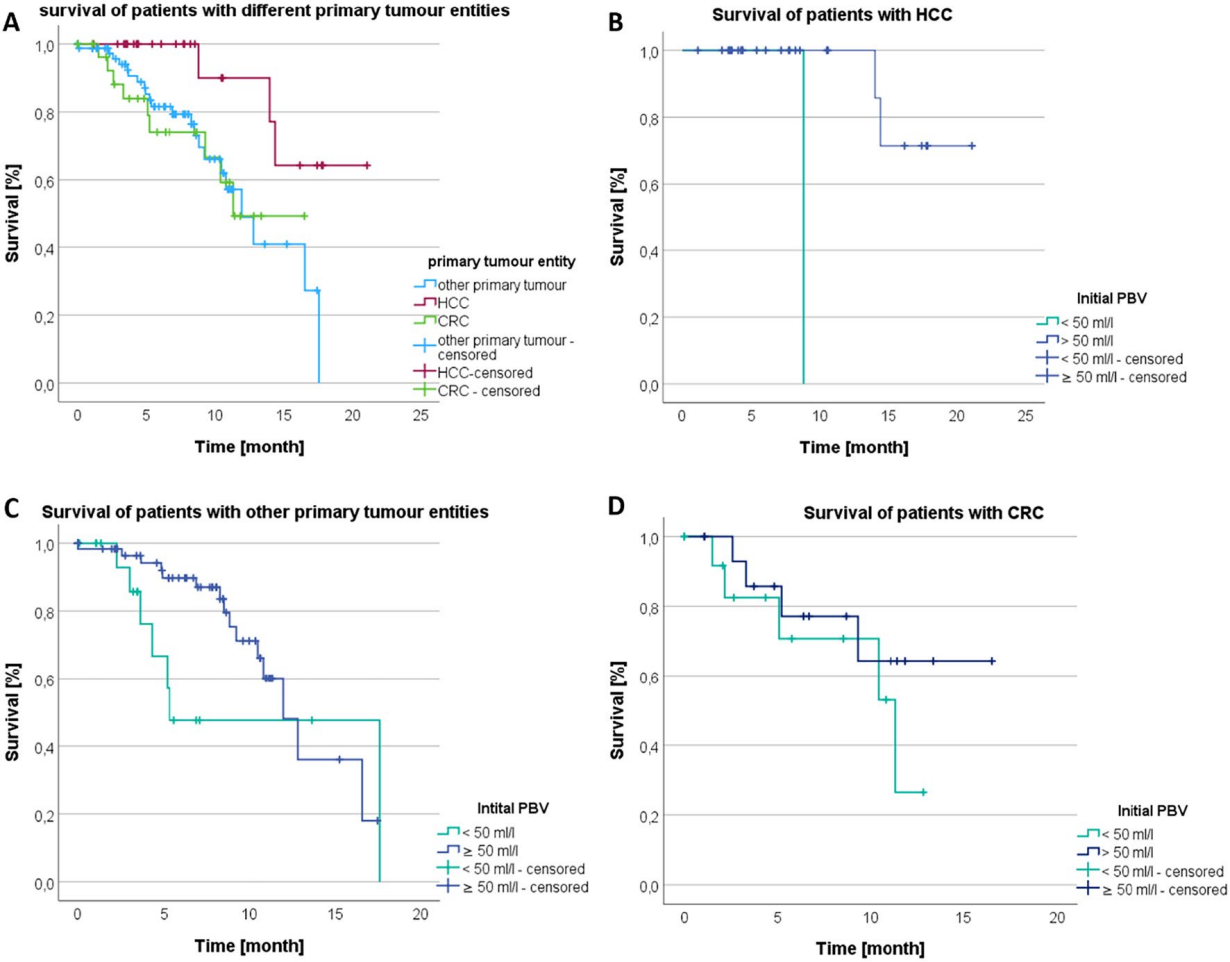
**A** Survival rate (%) related to time in month for different tumor entities.** B** Survival of patients with HCC divided in the two subgroups initial PBV < 50 ml/l and ≥ 50 ml/l. **C** Survival of patients with other primary tumor divided in the two subgroups initial PBV < 50 ml/l and ≥ 50 ml/l. **D** Survival of patients with CRC divided in the two subgroups initial PBV < 50 ml/l and ≥ 50 ml/l

The overall survival time was also analyzed in dependence of the different primary tumor entities: HCC, CRC, and other primary tumors. Patients with HCC (*n* = 27) showed an average survival time of *M* = 18.09 and thus survived longer than patients with CRC (*n* = 30, *M* = 11.64) or with other primary tumors (*n* = 80, *M* = 12.03, Fig. 6A). The difference between HCC and CRC was significant (*p* = 0.020), also the difference between HCC and other primary tumors (*p* = 0.008). On the other hand, there was no significant difference between CRC and other primary tumors (*p* = 0.850).

Additionally, the effect of PBV on survival since 1st TACE was investigated separately for HCC, CRC, and other primary tumors (Fig. 6B–D). Median survival indicated that in the HCC group survival was longer at PBV ≥ 50 ml/l with *M* = 19.12 months than in the group PBV < 50 ml/l with *M* = 8.82 months. Only the HCC group with an initial high PBV volume was there a significant association with prolonged survival with *p* = 0.003. For CRC it was *M* = 9.18 months for PBV < 50 ml/l and *M* = 12.69 months for PBV ≥ 50 ml/l. For other primary tumors it was *M* = 12.21 months for PBV < 50 ml/l and *M* = 10.51 months for PBV ≥ 50 ml/l. Thus, in none of the other two groups was there a significant correlation between initial PBV and prolonged survival with *p* = 0.345 for CRC and *p* = 0.163 for other primary tumors.

## Discussion

The increase in life expectancy and advancements in diagnostic procedures have led to a rise in malignant diseases, including various types of cancer [4]. The increase in life expectancy and advancements in diagnostic procedures have led to a rise in malignant diseases, including various types of cancer [4]. Consequently, the demand for dependable biomarkers and measurable parameters for assessing disease severity and therapy progress is also on the rise. To address these demands, research into disease progression, cellular mechanisms, and diagnostic options is just as crucial as the exploration of existing parameters and their predictive capabilities.

This study focused on patients who have HCC, CRC, or other primary tumor entities which were treated by TACE. The aim was to investigate whether a high initial PBV value is associated with a better treatment response and prolonged survival. Current gold standard in the assessment of tumor treatment success are the Response Evaluation Criteria in solid tumors, the so called RECIST criteria [3, 18]. These criteria are based on a 2-dimensional measurement of the tumor either by MRI or by spiral CT, where the longest diameter of solid tumor lesions is assessed and used as comparison value after the intervention. Hence, the tumor shrinkage is the only treatment parameter which is focused on [12] and the measurement is followed by an internationally valid classification in partial response (PR), complete response (CR), progressive disease (PR), and stable disease (SD) [18, 26]. This system makes it possible to evaluate the therapy progress in a standardized manner but also has some limitations based on the diversity of treatment options and cancer types [6]. The traditional RECIST criteria and thresholds of response were developed in order to evaluate the response to cytotoxic chemotherapeutic treatments and modern personalized medical approaches, which have other outcomes than tumor shrinkage, may not fit in this criteria [6]. Additionally, liver lesions and their treatment push the RECIST criteria to their limits based on the alterations of visibility and size of liver tumors after contrast injection depending on time, treatment and devascularization and also morphological changes of the lesions and scarring after focal therapy such as chemoembolization [6]. For patients with these lesions the criteria were adapted and the mRECIST (modified RECIST) criteria were established in 2010; these also address changes in the viable portion and tumor necrosis in order to evaluate the effectivity of focal therapies [6] and they are taken into account in the European guideline [7].

These criteria and specific diagnostic procedures open up the possibility to evaluate therapy success of TACE in HCC patients. One of those diagnostic methods is the C-arm CT which allows the visualization of the vascularity of the tumor tissues and thus, the measurement of the PBV.

Previous studies indicate that PBV measurements can assess TACE treatment success and that there is a correlation between a high initial PBV value and tumor response [9, 10, 15, 16, 24]. The results of the studies mentioned can be supported by comparable results of our study. We are able to show that there is a significantly higher decrease in PBV and tumor size after TACE with a high initial PBV, than with a low initial PBV. Similar results were reported by Vogl, Schäfer et al. who showed in a comparable study that in patients with a high initial PBV > 100 ml/l the percental decrease in PBV and tumor size is greater than with a low initial PBV < 50 ml/l [25]. The interesting question now is whether a high initial PBV value is associated with prolonged survival. To the best of the author's knowledge, there are currently no comparable studies on survival in relation to the initial PBV value. In our work, we refer to the study of Syha, who mentioned in his work that future survival analyzes should be performed in relation to the initial PBV value [17].

Our survival analysis results indicate that patients with high initial PBV survived significantly longer than patients with low initial PBV (*p* = 0.002)—on average nearly 5 months longer. However, the subgroup analysis of HCC, CRC, and patients with other primary tumors showed only that a higher initial PBV significantly increased the survival rate for HCC patients. For the other groups we found that the survival time for high and low PBV was not significantly different, which may be due to the small patient population. It is therefore advisable to expand the patient population in future studies and to conduct studies over a longer observational period. In future studies, expanding the patient population and conducting investigations over an extended observational period, while considering the diverse tumor burdens, will provide a more comprehensive understanding of the relationship between initial PBV values, treatment responses, and overall survival across various malignancies. With regard to the detailed analysis of differences between groups in terms of tumor biology or other baseline characteristics, we would like to emphasize that consideration of additional parameters such as ECOG status would have further reduced the size of our patient cohorts. Given the already limited sample size, smaller subgroups could have led to statistical uncertainty and limited interpretability.

Our aim was to include patients receiving TACE under as comparable conditions as possible in their respective groups. However, due to the complexity and time involved in the detailed inclusion of parameters like ECOG status, it was not feasible for us to make these allocations at this stage. This could represent a promising perspective for future studies specifically targeting the collection and analysis of such baseline characteristics. We acknowledge the importance of more comprehensive consideration of such factors and could view this as a suggestion for further research.

The results could indicate that a high PBV value was associated with prolonged survival and patients with HCC could possibly benefit from better prognosis estimation and potential extended lifetime with improved quality of life. Further investigations are necessary to evaluate the predictive power of the initial PBV in patients with malignant liver tumors before PBV can be used as a prognostic marker in diagnostics. In our discussion, we can underscore the significance of our findings regarding the elevated PBV in more vascular tumors in relation to overall survival. This outcome implies that tumors with a robust vascular supply may exhibit a heightened responsiveness to Transarterial Chemoembolization (TACE), potentially due to their greater dependence on arterial blood supply. Consequently, TACE might be more efficacious in treating such tumors, ultimately leading to prolonged survival.

Additionally, it would be pertinent to consider future investigations exploring whether analogous correlations hold true for alternative treatment modalities, such as radiation therapies or surgical resections. Such research endeavors could contribute to a comprehensive understanding of PBV's role as a prognostic factor across diverse therapeutic approaches and potentially assist in the optimal selection of treatment options tailored to specific tumor types.

Limitations of this approach to establish PBV as a biomarker were the time consuming preparation and analysis of PBV cards (it took an average of 15 min to create one PBV card), which is a disadvantage in the daily clinical life. All patients in this study received TACE on Syngo Artis Pheno CT (second generation) and/or Syngo Artis Zeego (first generation) and an impact on the results based on the type of device cannot be excluded. However, since all PBV cards, regardless of whether TACE was performed on 1st or 2nd generation devices, were created on the same workstation and all data were evaluated under the same conditions, this is unlikely. Furthermore, one should not disregard the heterogeneity of the patient collective. All patients with malignant liver disease and different primary tumors were included in this study. It must also be remembered that the primary tumors, underwent different pretreatments, some of which were not traceable. In some patients, the initial diagnosis was up to 20 years ago so that more detailed information on possible pretreatments were no longer accessible. An influence of these possible pretreatments on the results of our study is unlikely but cannot be ruled out completely. Additionally, theoretical considerations regarding potential effects of Lipiodol on the accurate calculation of PBV have been made. While Lipiodol is commonly used in transarterial chemoembolization (TACE) procedures and plays a crucial role in the delivery of therapeutic agents to the tumor site, its presence could introduce complexities in the accurate calculation of parenchymal blood volume (PBV). Theoretical considerations and potential interactions between Lipiodol and imaging techniques may impact the precision of PBV measurements. It is essential to acknowledge the possibility of such effects on PBV calculations and consider them as potential sources of error in our study. Future research could delve into the specific influences of Lipiodol on PBV determination to enhance the accuracy of these measurements in the context of TACE interventions. In our study, we ensured that all patients underwent TACE treatment to an angiographic endpoint of stasis or near stasis. Efforts were made to ensure that embolization was as effective as possible in achieving maximum tumor necrosis. However, lesser reductions in PBV cannot be ruled out and could be attributed to various factors. One possible explanation for lesser PBV reductions could be tumor biology. Tumors are heterogeneous, and their blood supply can vary significantly. Some tumors may have extensive and complexly branched blood supplies that may not be entirely reached by TACE treatment, leading to lesser PBV reductions even when embolization is performed to the best of our abilities. Furthermore, suboptimal techniques or individual anatomical differences among patients could play a role. The precise placement of embolic material and vascular architecture can vary, affecting the efficiency of embolization and the resulting PBV reduction. It's important to note that further research is needed to better understand these issues and the reasons for varying PBV reductions after TACE. This could help enhance the effectiveness of TACE treatment and improve the accuracy of predicting treatment responses.

## Conclusion

In summary, we were able to show that the initial PBV in tumors of HCC patients can be associated with the survival rate of the patients and that patients with a high initial PBV (≥ 50 ml/l) responded better to TACE and show a significantly increased survival time and even a greater reduction of tumor volume compared with patients with lower initial PBV (< 50 ml/l). For other primary tumors or patients with CRC as primary tumor entity, the survival time was not significantly different between patients with high or lower initial PBV. Further investigations are needed to demonstrate the predictive power of initial PBV values for malignant liver tumors.

### Supplementary Information

Below is the link to the electronic supplementary material.

Supplementary file1 (DOCX 136 kb)

## Abbreviations

PBV: Parenchymal blood volume
TACE: Transarterial chemoembolization
HCC: Hepatocellular carcinoma
CRC: Colorectal carcinoma
CA: Carcinoma
MRI: Magnetic resonance imaging
CT: Computer tomography
MWA: Microwave ablation
RFA: Radiofrequency ablation
A: Arteria
LITT: Laser induced thermotherapy
M: Month
A: Average
BCLC: Barcelona Clinic Liver Cancer
CR: Complete response
SD: Stable disease
PD: Progressive disease
PR: Partial response
