# Supplementary material for: Baseline parenchymal blood volume is a potential prognostic imaging biomarker in patients with malignant liver tumors treated with transarterial chemoembolization
Source: Abdom Radiol (NY). 2024 Apr 20;49(9):3056–68. doi: 10.1007/s00261-024-04240-9 (PMC11335802; doi:10.1007/s00261-024-04240-9)
Supplement: Supplementary file 1 — Supplementary file1 (DOCX 136 kb) [file 261_2024_4240_MOESM1_ESM.docx]

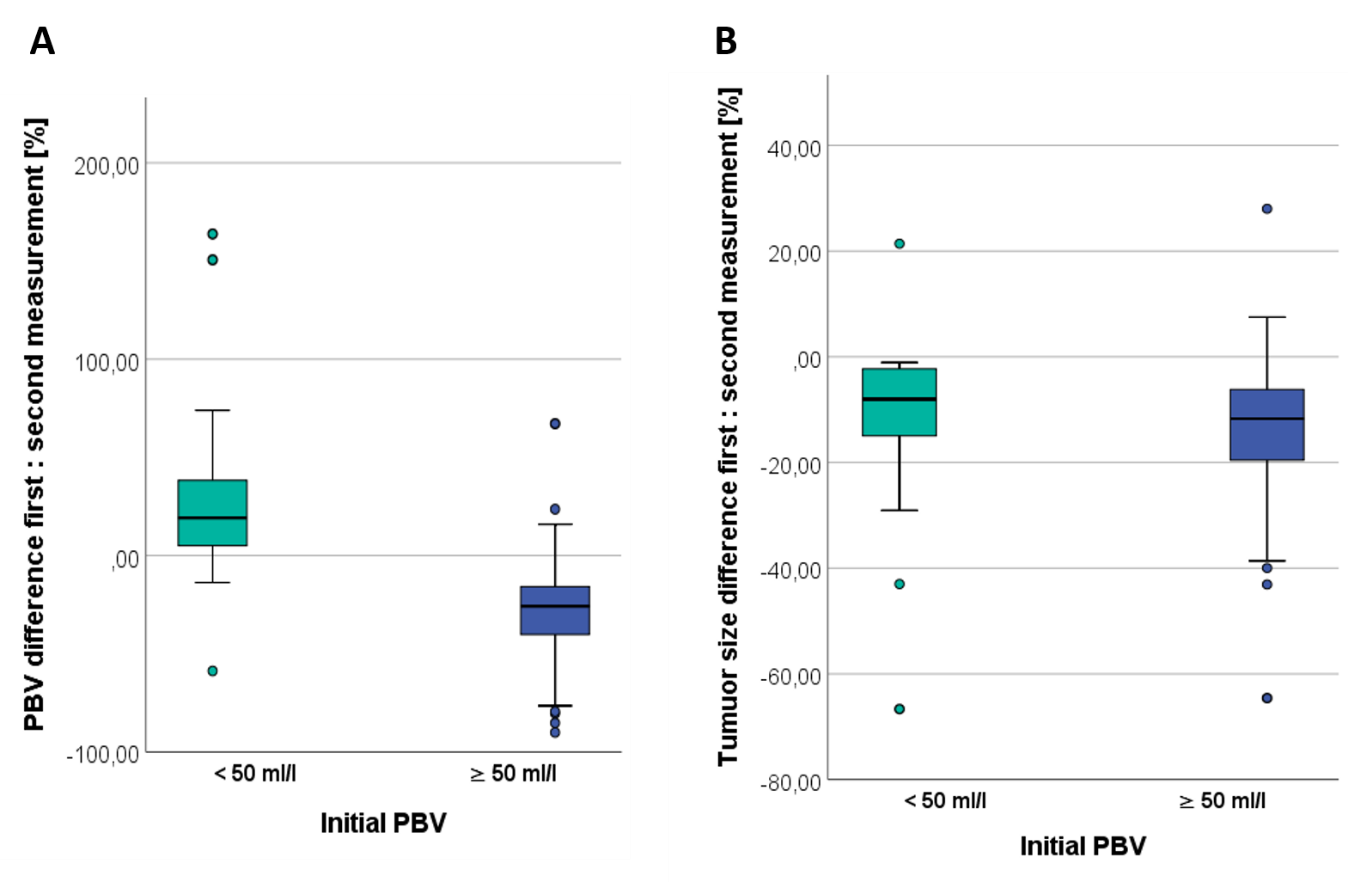


**Supplement Figure 1:** Boxplot (**A)** PBV differences first to second measurement [%] (**B**) tumour size difference first to second measurement [%].
